# PEDOT‐Integrated Fish Swim Bladders as Conductive Nerve Conduits

**DOI:** 10.1002/advs.202400827

**Published:** 2024-06-17

**Authors:** Hui Zhang, Dongyu Xu, Bin Zhang, Xiaofan Li, Minli Li, Chen Zhang, Huan Wang, Yuanjin Zhao, Renjie Chai

**Affiliations:** ^1^ State Key Laboratory of Digital Medical Engineering Department of Otolaryngology Head and Neck Surgery Zhongda Hospital School of Life Sciences and Technology Advanced Institute for Life and Health Jiangsu Province High‐Tech Key Laboratory for Bio‐Medical Research Southeast University Nanjing 210096 China; ^2^ Co‐Innovation Center of Neuroregeneration Nantong University Nantong 226001 China; ^3^ State Key Laboratory of Digital Medical Engineering School of Biological Science and Medical Engineering Southeast University Nanjing 210096 China; ^4^ Beijing Key Laboratory of Neural Regeneration and Repair Capital Medical University Beijing 100069 China; ^5^ The Eighth Affiliated Hospital Sun Yat‐sen University Shenzhen 518033 China; ^6^ Department of Rheumatology and Immunology Nanjing Drum Tower Hospital School of Biological Science and Medical Engineering Southeast University Nanjing 210096 China; ^7^ Department of Neurology Aerospace Center Hospital School of Life Science Beijing Institute of Technology Beijing 100081 China; ^8^ Department of Otolaryngology Head and Neck Surgery Sichuan Provincial People's Hospital University of Electronic Science and Technology of China Chengdu 610072 China; ^9^ Institute for Stem Cell and Regeneration Chinese Academy of Science Beijing 100101 China; ^10^ Southeast University Shenzhen Research Institute Shenzhen 518063 China

**Keywords:** conductivity, fish swim bladder, nerve conduit, nerve regeneration, PEDOT, topography

## Abstract

Advanced artificial nerve conduits offer a promising alternative for nerve injury repair. Current research focuses on improving the therapeutic effectiveness of nerve conduits by optimizing scaffold materials and functional components. In this study, a novel poly(3,4‐ethylenedioxythiophene) (PEDOT)‐integrated fish swim bladder (FSB) is presented as a conductive nerve conduit with ordered topology and electrical stimulation to promote nerve regeneration. PEDOT nanomaterials and adhesive peptides (IKVAV) are successfully incorporated onto the decellularized FSB substrate through pre‐coating with polydopamine. The obtained PEDOT/IKVAV‐integrated FSB substrate exhibits outstanding mechanical properties, high electrical conductivity, stability, as well as excellent biocompatibility and bioadhesive properties. In vitro studies confirm that the PEDOT/IKVAV‐integrated FSB can effectively facilitate the growth and directional extension of pheochromocytoma 12 cells and dorsal root ganglion neurites. In addition, in vivo experiments demonstrate that the proposed PEDOT/IKVAV‐integrated FSB conduit can accelerate defective nerve repair and functional restoration. The findings indicate that the FSB‐derived conductive nerve conduits with multiple regenerative inducing signals integration provide a conducive milieu for nerve regeneration, exhibiting great potential for repairing long‐segment neural defects.

## Graphical abstract

A type of PEDOT‐integrated fish swim bladder is proposed as a conductive nerve conduit with anisotropic surface topography, electrical conductivity, flexibility, and biological cues for peripheral nerve regeneration. PEDOT nanoparticles and IKVAV peptides are immobilized on the fish swim bladders, thus promoting the growth and directional extension of neural cells, together with accelerating regeneration and functional recovery of damaged nerves.

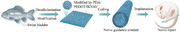

## 1 Introduction

Nerve injuries from traumas, including traction, ischemic and crush injuries, generally cause significant morbidity in patients.^[^ ^1^ ^]^ Recently, multifarious nerve conduits derived from natural and/or synthetic polymers have been exploited to treat nerve injuries.^[^ ^2^ ^]^ These neural conduits have the ability to serve as functional bridges for axon extension and directional neuron outgrowth, as well as provide a suitable microenvironment for nerve regeneration.^[^ ^3^ ^]^ To further improve their performance, conductive elements such as metal particles and carbon‐based materials have been integrated into nerve conduits to promote cellular activities.^[^ ^4^ ^]^ Although with many successes, the majority of conduits request time‐consuming synthesis procedures while accompanied by the absence of surface morphology, leading to restricted outcomes. In addition, difficult degradation and controversial biocompatibility are often problems for these integrated conductive materials. Therefore, new conductive nerve conduits with topological structures and satisfactory biological properties are still anticipated for injured nerve regeneration.

**Figure 1:**
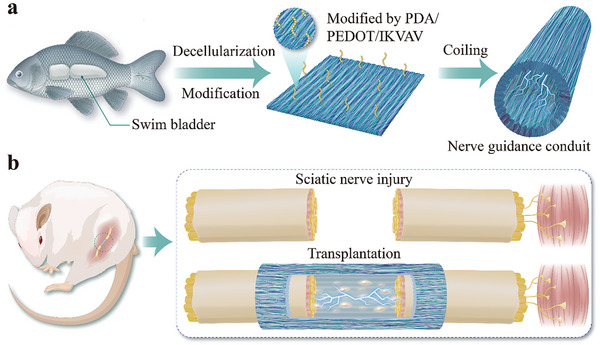
Schematic diagram of PEDOT‐integrated fish swim bladders as a conductive nerve conduit for peripheral nerve repair. a) The preparation process of PEDOT‐integrated fish swim bladder conduit. b) Scheme of conductive nerve conduit applying for sciatic nerve repair.

Herein, we present novel poly(3,4‐ethylenedioxythiophene) (PEDOT)‐integrated fish swim bladders (FSBs) as conductive nerve conduits to promote peripheral nerve repair, as schemed in **Figure** **1**. FSB is a naturally occurring organ that helps fish float freely in water and consists of 80% type I collagen fibers.^[^ ^5^ ^]^ Benefitting from its abundance, affordability, low antigenicity and allergenicity, FSB has a wide range of potential applications in tissue engineering.^[^ ^6^ ^]^ Recent research has also revealed that FSB is a fantastic choice for guided tissue scaffolds due to its globally aligned collagen fibers organization and similar matrix composition to natural tissues.^[^ ^7^ ^]^ However, microenvironments established solely by topological matrices usually constrain cell binding sites and electrical signal transmission, thereby being limited in promoting cell attachment and proliferation.^[^ ^8^ ^]^ In contrast, as a polythiophene derivative, PEDOT exhibits high electrochemical property and stability, as well as satisfactory biocompatibility, which has been widely used in tissue regeneration like nerve tissue repair and bone regeneration.^[^ ^9^ ^]^ Thus, we conceive that the integration of PEDOT into FSB will create a brand‐new nerve conduit for injured nerve repair.

In this paper, we developed the desired PEDOT‐composed FSB conduits with good flexibility, conductivity, anisotropic surface morphology and biological cues for peripheral nerve regeneration. During this process, a highly anisotropic topological substrate was prepared based on natural *Carassius auratus* FSB, followed by in situ polymerization of PEDOT nanoparticles and isoleucine‐lysine‐valine‐alanine‐valine (IKVAV, a sequence of laminin) immobilization. Benefiting from the tight coverage of the conductive coating, the composite substrates not only retained the topology of the natural FSB but also inherited the high conductivity from PEDOT, thereby improving the electrical signal transmission between cells and enhancing the outgrowth, extension and orientation of nerve cells. In addition, IKVAV enabled the FSB substrates to be more biocompatible and have abundant cell adhesion sites. Further rat sciatic nerve defect models demonstrated that the proposed PEDOT/IKVAV‐integrated FSB conduit could accelerate defected nerve regeneration and functional recovery. These results indicate that our conduits, integrating high topology with electrical stimulation and biological cues, will be a viable option for promoting peripheral nerve repair in the clinic.

## 2 Results and Discussion

**Figure 2:**
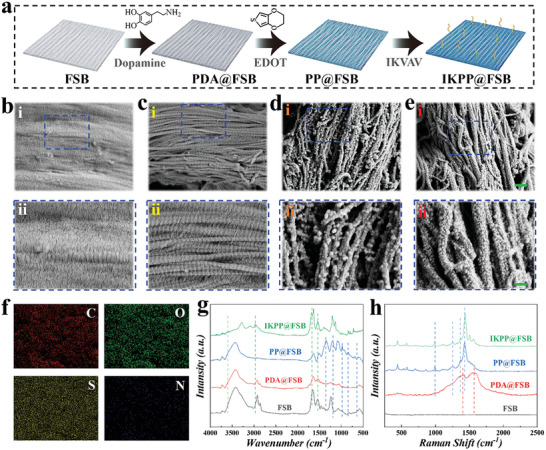
Characterization of composite FSB. a) Fabrication process of FSB with different treatments. b–e) SEM images of the natural FSB, decellularized FSB, PDA@FSB, and PP@FSB. Scale bars are 1 µm and 200 nm in i and ii, respectively. f) EDS mapping of PP@FSB. g) FTIR spectra of different substrates. h) Raman spectra of different substrates.

In a typical experiment, natural FSB was collected from fresh fish and treated with multiple procedures, as illustrated in **Figure** **2**a. Before surface modification, we utilized various decellularization methodologies, including freeze‐thaw cycles, sodium deoxycholate (SDC), as well as DNase and RNase, to disrupt genetic material and cellular debris from the crude tissue. Consequently, the resultant FSB hydrogel contained nearly no immunogenic cellular remnant, as demonstrated by Hematoxylin‐eosin (H&E) staining and DNA content analysis (Figures S1 and S2, Supporting Information); meanwhile, the native structures of FSB were maintained completely. As shown in Figure 2b,c, decellularized FSB exhibited an anisotropic ordered arrangement of collagen nanofibers, while undecellularized FSB (UD‐FSB) showed a clean surface without topological structures, further demonstrating the successful decellularization of native swim bladder tissue.

In recent decades, the mussel‐inspired modification strategy has provided an effective and easy way to combine various functional materials, such as bioactive molecules and conductive elements.^[^ ^10^ ^]^ Thus, we constructed a polydopamine (PDA) coating by in situ polymerization, and scanning electron microscopy (SEM) images confirmed the uniform deposition of PDA nanoparticles (Figure 2d). Then, the conductive FSB hydrogel was obtained through covalent cross‐linking of the 3,4‐ethylenedioxythiophene (EDOT) monomer at various concentrations onto the FSB hydrogel. As shown in Figure 2e, the generated PEDOT nanoparticles were evenly dispersed on the collagen nanofibers, forming a well‐aligned conductive network. Meanwhile, the sulfur (S) element in the energy dispersive spectrometer (EDS) analysis image validated the successful attachment of PEDOT nanoparticles to the FSB nanofibers (Figure 2f). Additionally, we modified PEDOT‐coated FSB (PP@FSB) with IKVAV to improve cell adhesion and neurite growth. As demonstrated in Figure S3 (Supporting Information), a fluorescent signal was observed on the IKVAV‐immobilized PP@FSB (IKPP@FSB), whereas no fluorescent signal was seen on PP@FSB. Then, we conducted Fourier transform infrared spectroscopy (FITR) and Raman analysis for further component confirmation. The FTIR spectrum of PP@FSB exhibited absorption bands at 1350 cm^−1^, corresponding to C─C stretching, 1200 cm^−1^ for C─O─C peak stretching vibration, as well as 980, 844, and 692 cm^−1^ for stretching of the C─S bond in the thiophene ring (Figure 2g). After IKVAV immobilization, the intensity of the adsorption peak at 1541 cm^−^^1^ was evident due to the N─H bending of IKVAV. Raman spectra displayed that the stretching and deformation of the aromatic rings of the two peaks at 1402 and 1587 cm^−^^1^ were strong evidence of PDA presence (Figure 2h). Besides, the peaks of 1428, 1369, and 1252 cm^−1^ were related to the C═C symmetrical stretching of the thiophene ring, C─C intra‐thiophene ring, and C─C inter‐ring stretching vibrations, respectively. The vibrational mode at 994 cm^−^^1^ was allocated to the oxyethylene ring deformation in the PEDOT chains.

In order to verify the stability of the substrate in the wet environment, we tested the dynamic swelling ratio of FSB, PDA@FSB, and PP@FSB in PBS. As shown in Figure S4 (Supporting Information), all materials reached swelling equilibrium at ≈1.5 h, and the swelling ratio remained relatively stable since then. Obviously, the swelling ratio of PP@FSB was significantly lower than that of FSB and PDA@FSB, which was attributed to the doping of PEDOT nanoparticles. As nerve scaffolds are required to be degradable, we further explored the degradation rate of FSB, PDA@FSB, and PP@FSB under different conditions. As shown in Figure S5a (Supporting Information), unmodified FSB was utterly degraded in PBS, 2, 5, and 20 U mL^−1^ collagenase solutions after 37, 30, 25, and 15 days. PDA@FSB was completely degraded after 63 and 42 days in the 5 and 20 U mL^−1^ collagenase solution, while the quality ratio dropped to 25.832 ± 5.089% and 15.882 ± 2.851% after 70 days under the conditions of PBS and 2 U mL^−1^ collagenase solution (Figure S5b, Supporting Information). In contrast, PP@FSB showed a slow degradation rate (Figure S5c, Supporting Information). After being soaked in PBS, 2, 5, and 20 U mL^−1^ collagenase solutions for 70 days, the mass of PP@FSB dropped to 83.903 ± 4.404%, 78.170 ± 2.703%, 70.089 ± 2.746%, and 62.948 ± 3.406% of the original mass, respectively. Resultantly, PEDOT doping greatly delayed FSB degradation, beneficial for long‐term use in the body.

**Figure 3:**
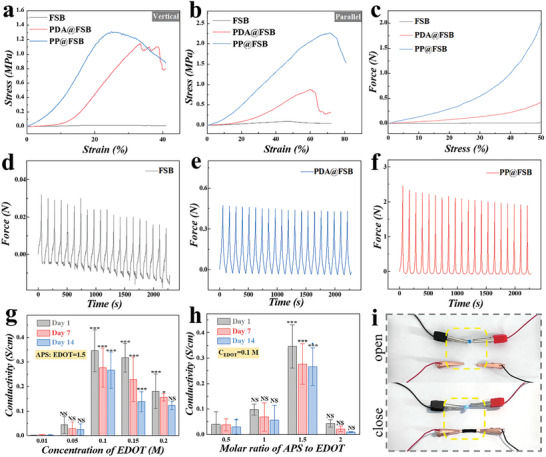
Evaluation of mechanical and conductive properties of composite FSB. a,b) Tensile stress–strain test in the alignment fibers vertical (a) and parallel (b) orientations. c) Compressive stress–stain curves of different scaffolds. d–f) Cyclic stress test. g,h) Influence of EDOT (g) and APS (h) contents on the conductivity of PP@FSB, as well as the conductive stability of PP@FSB immersed in PBS solution. i) LED bulb illuminance pictures.

Due to the heterogeneous microstructure present inside FSB, the mechanical properties of FSB hydrogel were investigated in both vertical and parallel directions. As seen in **Figure** **3**a,b, when stretched parallel (//) to the aligned fibers, the FSB, PDA@FSB, and PP@FSB hydrogels behaved more stiffly compared to perpendicular (⊥) stretch to the collagenous fibers, which could be ascribed to uniaxial reinforcement by aligned nanofibers. It was observed that PP@FSB hydrogels had improved resilience (in the two directions) compared to that of FSB and PDA@FSB hydrogels, which revealed that PEDOT modifications rendered FSB stronger (Figure S6, Supporting Information). Besides, PP@FSB conduits showed improved compressed strength compared with others, as shown in Figure 3c. The mechanical resilience of various conduits was evaluated through repeated compressive cycles (Figure 3d‐f). Following 20 compressive cycles, naked FSB conduits experienced a progressive loss of mechanical strength. Conversely, PDA@FSB and PP@FSB conduits displayed substantial compressive stress levels during cyclic compression. The physical properties allow the neural scaffold to resist structural damage caused by the multiple mechanical deformations it undergoes after implantation, providing long‐term support and guidance for nerve growth.

Of note, the influence of monomer EDOT and an oxidizing agent (ammonium persulfate, APS), as well as PDA coating, on the conductivity of the PP@FSB were studied. As observed in Figure S7 (Supporting Information), PDA pre‐modification significantly improved the conductivity of PP@FSB, wherein the conductivity of PP@FSB was ≈72 times higher than that of the directly PEDOT‐coated FSB without PDA coating. Besides, when the ratio of APS: EDOT was set as 1.5 and the concentration of EDOT was 0.1 m, the PP@FSB showed the highest conductivity (Figure 3g,h). After that, the obtained PP@FSBs with different contents of APS and EDOT were soaked in PBS buffer solution and placed on a shaker, and the solution was collected and photographed on the 7th and 14th day, respectively. As exhibited in Figure S8 (Supporting Information), when the monomer concentration exceeded 0.1 m, a large number of PEDOT nanoparticles fell away from the PP@FSB two weeks later, resulting in a decline in its conductive property. Therefore, excessive EDOT may lead to weak bonding of the conductive layer, ultimately resulting in the decrease of PP@FSB conductivity. According to the above results, 0.1 m was selected as the final reaction concentration of the EDOT monomer, while the APS: EDOT ratio was set at 1.5. In addition, when being rolled into a conduit, the PP@FSB maintained its electrical conductivity, enabling the illumination of an LED bulb (Figure 3i).

**Figure 4:**
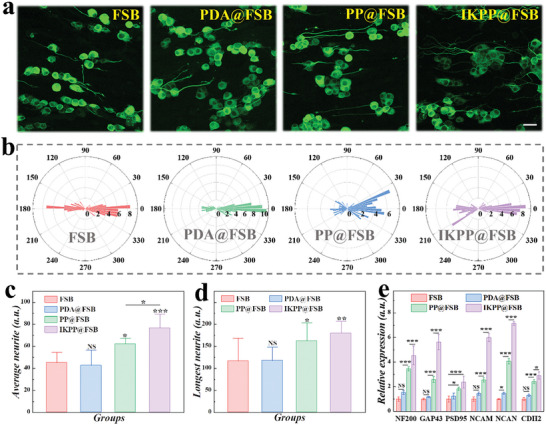
PC12 cells grown on different FSB substrates. a) Representative images of PC12 cells cultured on FSB, PDA@FSB, PP@FSB, and IKPP@FSB. Scale bar is 20 µm. b) Polar histograms of neurites of PC12 cells cultured on FSB, PDA@FSB, PP@FSB, and IKPP@FSB. c,d) Statistical analysis of average (c) and longest (d) neurite lengths of PC12 cells cultured on FSB, PDA@FSB, PP@FSB, and IKPP@FSB. e) Relative gene expression of PC12 cells cultured on FSB, PDA@FSB, PP@FSB, and IKPP@FSB.

To assess the biological properties of the obtained FSB substrates, pheochromocytoma 12 (PC12) cells, dorsal root ganglion (DRG) neurons, and Schwann cells (SCs) were seeded on different substrates, and the cytotoxicity was first determined by Cell Counting Kit 8 (CCK‐8 kit) (Figure S9, Supporting Information). After culturing for five days on the FSB, PDA@FSB, and PP@FSB, PC12 cells of all groups possessed similar proliferation rates to tissue culture polystyrene (TCP), indicating the excellent cytocompatibility of prepared FSB and modified substrates. Besides, SCs and DRG neurons seeded on different substrates for three days showed similar viability, further confirming the friendliness of FSB with or without modification on primary cells (Figures S9b,c and S10, Supporting Information). Furthermore, highly differentiated PC12 cells with multiple processes like neuron axons were applied to assess the directional guidance of the topological structures of swim bladder samples to cells. As exhibited in Figure S11 (Supporting Information), highly differentiated PC12 cells exhibited neurites elongated along the nanofibers of naked and modified FSB while randomly distributed on the TCPs after culturing for two days. Besides, undifferentiated PC12 cells with round shapes were utilized to evaluate the effect of conductivity and biological cues on cellular growth and differentiation due to their high sensitivity to external stimuli. As shown in **Figure** **4**a,b, the neurites of PC12 cells grown on different FSB substrates extended along the nanofibers in a direction. In addition, PC12 cells in the PP@FSB and IKPP@FSB groups displayed longer neurites than those in the TCP, FSB, and PDA@FSB groups, demonstrating improved cell differentiation (Figure 4c,d).

To further gain insight into cell‐substrate interactions at the molecular level, we utilized real‐time fluorescent quantitative polymerase chain reaction (RT‐qPCR) to detect gene expression of multiple critical cell behavior‐related factors, including neurofilament protein 200 (NF200), postsynaptic density protein 95 (PSD95), growth‐associated protein 43 (GAP43), neural cell adhesion molecule (NCAM), neurocan (NCAN), and cadherin 2 (CDH2) (Figure 4e). In the PP@FSB and IKPP@FSB groups, the expression of axon‐related proteins (NF200, GAP43, and PSD95) was higher than that of the FSB and PDA@FSB groups. Besides, the expression of adhesion regulators NCAM, NCAN, and CDH2 was significantly upregulated in the PP@FSB and IKPP@FSB groups compared to the FSB and PDA@FSB groups. Additionally, NCAM, NCAN, and CDH2 levels on the IKPP@FSB substrate were higher than those on PP@FSB without IKVAV modification. These results indicate that topological cues, conductivity, and biological cues played positive roles in guiding neurite extension, enhancing cell differentiation, and promoting cell adhesion.

**Figure 5:**
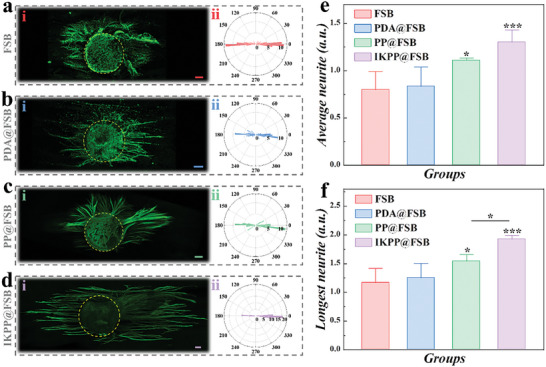
DRG grown on different FSB substrates. a–d) Immunofluorescent images of DRG cultured on different substrates. Scale bars are 100 µm in (a,b,d), and 50 µm in (c). e) The ratio of average neurite length to cell body diameter of each DRG cultured on FSB, PDA@FSB, PP@FSB and IKPP@FSB. f) The ratio of the longest neurite length to cell body diameter of each DRG cultured on FSB, PDA@FSB, PP@FSB, and IKPP@FSB.

Then, after being cultivated on the various substrates for four days, the directional neurite growth of DRGs was observed. It was discovered that the neurites protruded from DRGs and followed the direction of fiber alignment in all groups, as exhibited in **Figure** **5**a–d. The ratio of neurite length to cell body diameter of each DRG cultured on FSB, PDA@FSB, PP@FSB and IKPP@FSB was analyzed. As a result, the ratio of average neurite length to cell body diameter was 0.799 ± 0.158, 0.835 ± 0.139, 1.110 ± 0.084, and 1.302 ± 0.133, respectively, while the ratio of longest neurite length to cell body diameter was 1.167 ± 0.235, 1.252 ± 0.194, 1.541 ± 0.093, and 1.926 ± 0.222, respectively (Figure 5e,f). Therefore, modifying PEDOT and IKVAV improved the neurite outgrowth, indicating the positive effects of electrical conductivity and biological cues.

**Figure 6:**
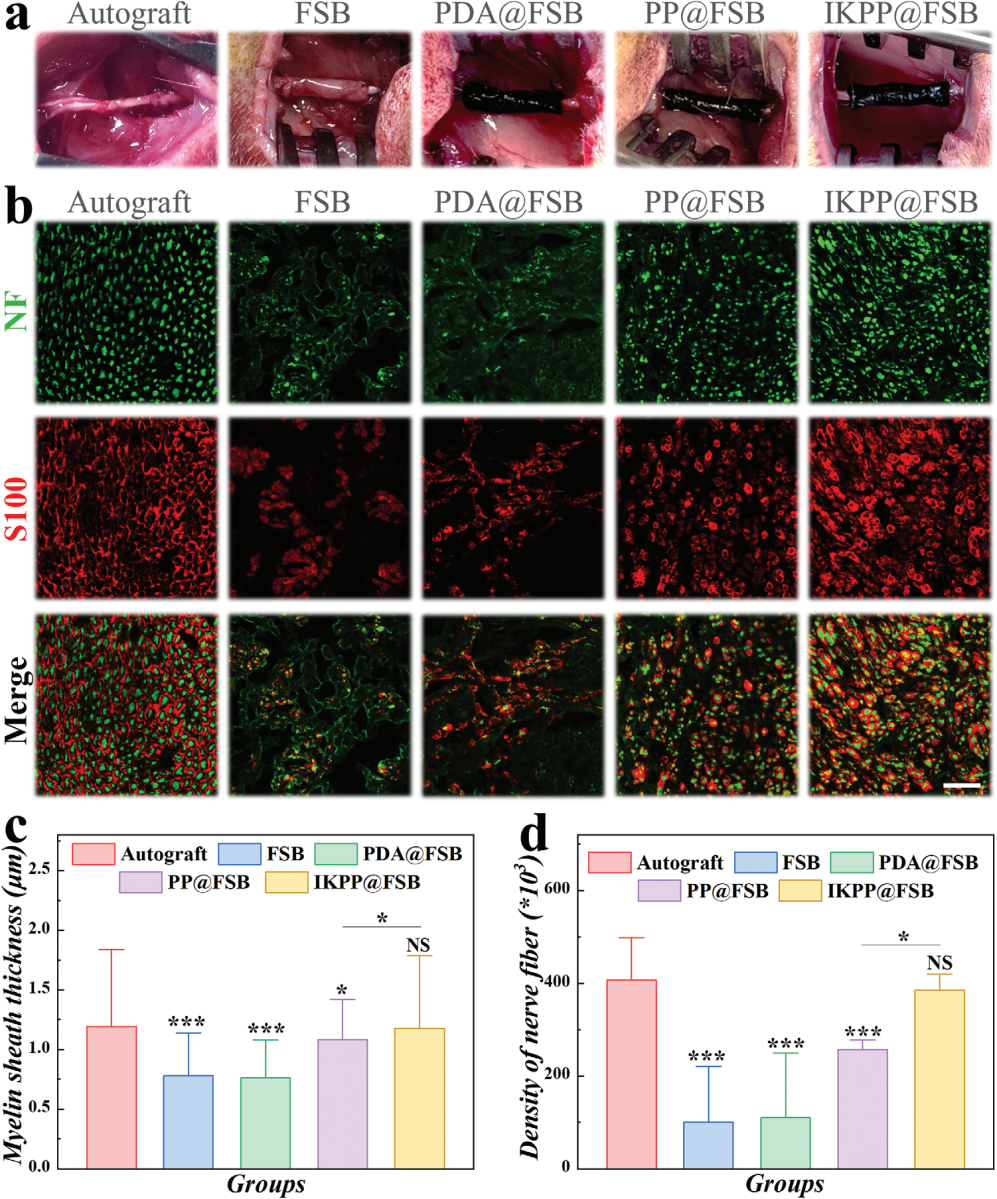
Sciatic nerve regeneration. a) Representative images of implant surgery. b) Immunofluorescent images of NF and S100‐positive nerves. Scale bar is 40 µm. c) Quantitative analysis of the myelin sheath thickness. d) Statistical analysis of nerve fiber density per square micron.

The in vivo study was conducted to assess the impact of the nerve conduit derived from the FSB on the repair of peripheral nerve damage. Prior to in vivo implementation, the FSB membrane was rolled into a nerve conduit using a glass capillary with a diameter of 1.5 mm and then secured with surgical sutures. Then, the conduits were utilized to connect the two ends of the sciatic nerve defect in male rats, followed by end‐to‐end suturing of the sciatic nerve stumps and the conduits (**Figure** **6**a). All rats were randomly divided into five groups, namely, Autograft, FSB, PDA@FSB, PP@FSB, and IKPP@FSB, wherein the rats treated with autologous transplantation were classified as the control group. Each group's nerve regeneration and functional recovery were evaluated two months after the surgical interventions for improved comparison (Figure S12, Supporting Information). It was observed that the scaffold material had an obvious degradation trend, but some remained after two months of treatment. Given that the repair period in long nerve truncation or even large animal models is lengthy, often requiring more than half a year of recovery time, it can be reasonably assumed that the currently observed degradation rate is in line with expectations. Besides, the regenerated nerve tissues of all five groups were sectioned longitudinally, and immunofluorescence staining was carried out using SCs marker S100 and axon marker NF. As shown in Figure S13 (Supporting Information), a substantial volume of myelinated nervous fibers was regenerated in the IKPP@FSB group. Conversely, the FSB and PDA@FSB groups showed severed and incomplete nerve fibers. Furthermore, the regenerated nerves were subsequently sectioned transversely (Figure 6b). It was demonstrated that the nerve fibers formed in the IKPP@FSB group were denser and featured thicker myelin sheaths, similar to those regenerated in the autologous transplantation group (Figure 6c,d). This outcome might be a result of the interplay between swim bladder topology, electrical conductivity and biological cues of IKPP@FSB conduits in peripheral nerve regeneration.

**Figure 7:**
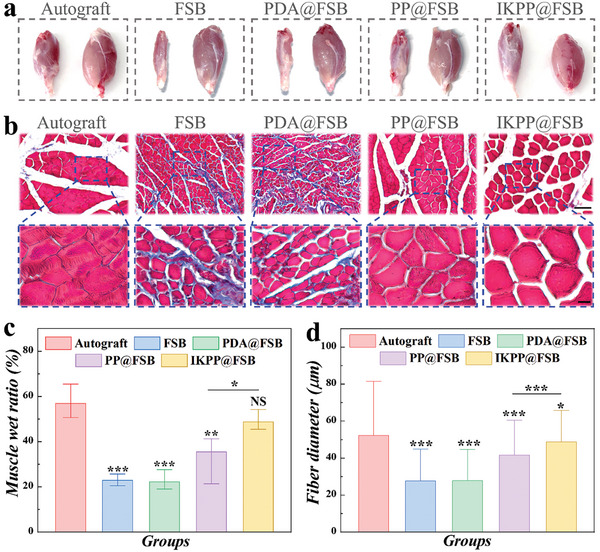
Sciatic nerve functional recovery. a) Optical images of gastrocnemius muscles of rats in the Autograft, FSB, PDA@FSB, PP@FSB, and IKPP@FSB groups. b) Representative Masson's trichrome staining of gastrocnemius muscles of rats in Autograft, FSB, PDA@FSB, PP@FSB, and IKPP@FSB groups. Scale bars are 100 and 20 µm. c) Statistical analysis of wet muscle ratios of different groups. d) Statistical analysis of muscle fiber diameters based on Masson's trichrome staining images.

An important indicator of an animal's sciatic functional recovery is the sciatic nerve functional index (SFI). On a scale from −100 to 0, 0 denotes normal function, while −100 represents utter loss of sciatic nerve function. We conducted a walking trajectory analysis and computed the SFI after two months of surgical implantation. The findings indicated that the IKPP@FSB group demonstrated superior functional recovery compared to the other three FSB‐derived conduit groups, approaching levels seen in the autograft group (Figure S14, Supporting Information). Besides, the gastrocnemius muscle serves as the terminal target tissue for sciatic nerve innervation. Two months after surgery, the bilateral gastrocnemius of rats in five groups was isolated, photographed, and measured. As seen in **Figure** **7**a, the rats in the PP@FSB and IKPP@FSB groups showed improved muscle atrophy compared to those in the FSB and PDA@FSB groups, suggesting that electrical conductivity and biological cues could significantly accelerate muscle recovery to varying degrees. In addition, the muscle wet‐weight ratio analysis of rats in different groups yielded consistent results (Figure 7c). Furthermore, Masson's trichrome staining of gastrocnemius muscle cross‐sections and counting of muscle fiber diameters revealed that rats in the PP@FSB and IKPP@FSB groups exhibited increased muscle fiber diameter, with IKPP@FSB being superior to the PP@FSB group (Figure 7b,d).

These results indicate that the concurrent application of topographic cues, peptides, and conductive materials can efficaciously stimulate the directional growth and differentiation of nerve cells in vitro and markedly enhance the regeneration rate, nerve function, and microstructure recovery of peripheral nerves in vivo. The integration of spatial topographic features and biological signals can synergistically mediate and improve the interaction between cells and implants, thereby providing a suitable microenvironment for tissue regeneration. In recent years, there has been a surge of interest in the introduction of bioactive materials in nerve tissue engineering, particularly those that are conductive and biological peptides, which is due to their active roles in various biological processes, including cell adhesion, proliferation, migration, differentiation, and others.^[^ ^11^ ^]^ For example, the combined use of carbon nanotube‐PEDOT conductive film and electrical stimulation promoted the growth of DRG neurites and myelination;^[^ ^12^ ^]^ polyaniline composite electrospun scaffolds enhanced the differentiation of neural stem cells into neurons.^[^ ^13^ ^]^ In addition, micro‐nanostructure design has been proven to be an efficacious strategy for regulating cellular behaviors such as directional cell migration, differentiation, and axon elongation. In light of these backgrounds, researchers have devised a plethora of topological structures like micro/nanofibers, micropatterns, micro/nanochannels, and so forth.^[^ ^14^ ^]^ For instance, the polypyrrole‐integrated micropatterned scaffold, as devised by Gomez et al., has been demonstrated to significantly promote the axon elongation of neurons.^[^ ^15^ ^]^ Similarly, the PEDOT/PSS film with grooves, as developed by Bianchi et al., has been shown to enhance neuronal axon elongation and development.^[^ ^16^ ^]^ Although these scaffolds have made significant progress in the field of nerve regeneration, their preparation often involves complex technical steps and may introduce toxic chemical reagents, which may cause toxic effects on organisms with toxic reagent residues. Consequently, it is of paramount importance to develop a nerve regeneration scaffold that is both efficacious and safe.

Our study employs an innovative approach to construct a novel PEDOT‐integrated FSB as a conductive nerve conduit. This scaffold exhibits excellent biological activity and conductive properties and makes full use of the biocompatibility and micro‐nano structural characteristics of natural FSB materials to create an ideal growth microenvironment for nerve cells. In comparison to existing technology, the preparation process of the scaffold in this study is straightforward and biosafe, without the necessity of doping with toxic chemical reagents. It has been demonstrated that the designed conductive FSB scaffold possessed satisfactory performance in peripheral nerve regeneration, which is at least in some respects comparable to microsurgical suturing of peripheral nerve ruptures. Additionally, this study provides new ideas for the development of neural tissue engineering scaffolds based on natural micro‐nano structural materials. Although this study yielded a series of promising findings, it is important to note that there are limitations. While the differences in nerve regeneration and functional recovery between different groups two months after surgery could be observed, in order to gain a more comprehensive understanding of the repair process, future studies should aim to increase observations at different time points after surgery. Besides, electrophysiological assessments may facilitate a more comprehensive evaluation of the regenerated nerves and innervated muscle function. Moreover, each factor's specific mechanisms of action in the repair of transected peripheral nerves must be further elucidated at the molecular level.

## 3 Conclusion

In summary, our proposed composite conductive topological nerve conduits possessed good flexibility, electrical conductivity, anisotropic surface morphology, and biological cues for peripheral nerve regeneration. By pre‐coating with PDA, PEDOT nanoparticles were successfully polymerized in situ on the decellularized FSB substrate and immobilization of IKVAV polypeptide was achieved. The prepared IKPP@FSB showed good biocompatibility, high conductivity and positive bioactivity, promoting the growth and directional extension of PC12 cells and DRG neurites. In vivo experiments showed that the developed IKPP@FSB nerve conduit could accelerate the regeneration and functional recovery of the defective nerve. These findings indicate that FSB‐derived nerve conduits with several regeneration‐inducing cues hold potential for nerve regeneration.

## 4 Experimental Section

### Materials

Fish swim bladders were obtained from the local market sources. EDOT, APS, Tris‐HCl, and dopamine were bought from Aladdin (China). The peptides IKVAV and FITC‐IKVAV were synthesized from Wuhan Haode Peptide (China). SDC was purchased from Sigma–Aldrich (USA), and CCK‐8 kit and phalloidin were bought from Beyotime (China). Hematoxylin‐eosin (H&E) staining kit and Masson's trichrome staining kit were bought from Solarbio (China).

### Decellularization of Fish Swim Bladder

First, the fat and blood tissues on the surface of the swim bladders were removed, and the swim bladders were washed three times with PBS. Subsequently, the swim bladders were subjected to a freeze‐thaw cycle by immersing them in liquid nitrogen and 37 °C ddH_2_O three times, followed by a 4‐hour wash with ddH_2_O at room temperature. Next, the swim bladders were treated with 4 wt.% SDC solution for 8 h. Post‐treatment, the swim bladders were rinsed with ddH_2_O for 30 min before the introduction of DNase and RNase, which were used to treat the swim bladders at 37 °C for an additional 4 h. Following enzymatic treatment, the swim bladders underwent ultrasonic disruption for 30 min. Finally, the swim bladders were washed with fresh PBS and stored at −20 °C for subsequent experimental use. The resulting acellular fish swim bladder was referred to as FSB.

### DNA Content Detection

After freeze‐drying, the swim bladder samples before and after decellularized treatment were collected. Then, these samples were cut into centrifugation tubes and weighed to a certain mass, wherein six replicate controls were set up. Next, the DNA component was extracted from the swim bladder samples according to the instructions of the DNA extraction kit (Tiangen, DP304).

### Surface Modification of FSB

The FSB was soaked in a solution of 2 mg mL^−1^ dopamine (Tris‐HCl, pH 8.0, 0.05 m) for 12 h. Following this incubation, the FSB was subjected to extensive washing with ddH_2_O to remove any unbound dopamine, resulting in the formation of PDA@FSB. This mixture was then placed on a shaking table for 30 min to ensure uniform distribution of the reagents. Thereafter, a solution of EDOT monomer was gradually added, initiating a polymerization reaction that lasted for 24 h. The FSB was resultantly modified with conductive polymer (PEDOT) after being washed with ddH_2_O, thus forming PP@FSB. Finally, the PP@FSB was immersed in a solution containing 50 µg mL^−1^ of the IKVAV polypeptide for 4 h at 37 °C in a constant temperature incubator, according to the previous study.^[^ ^11a^ ^]^ After cleaning with PBS, the swim bladder tissue coated with IKVAV was obtained and referred to as IKPP@FSB.

### Characterization

The structures and components of samples were analyzed by SEM, FTIR and Raman spectra. For SEM characterization, swim bladder samples were first fixed with Gluta fixative and pre‐cooled at 4 °C for 4 h. The samples were washed three times with PBS to fully remove the residual fixative and then dehydrated with 30%, 50%, 70%, 80%, 90%, and 100% ethanol in order, with each concentration being treated for 15 min. After drying at room temperature, the samples were cut into the appropriate size, fixed on the sample stage with conductive double‐sided tape, sprayed with gold nanoparticles on the sample surface for 2 minutes by plasma sputtering, and finally observed by SEM. For FTIR characterization, the freeze‐dried fish swim bladder samples were cut into pieces and ground with well‐dried potassium bromide powder, and the treated samples were obtained using a tablet press for FTIR analysis. As to Raman characterization, freeze‐dried fish swim bladders were ground into powder samples and measured through a Thermo Fischer DXR laser Raman spectrometer.

### Electrochemistry

The electrical conductivity of PP@FSB was tested by an electrochemical workstation. PP@FSB samples with different APS and EDOT contents were cut into rectangles and their conductivity was measured by selective cyclic voltammetry using an electrochemical workstation. In order to assess the stability of the conductive layer of PP@FSB, it was immersed in PBS for a fortnight, and the buffer was collected weekly in a transparent sample bottle and photographed to record the degree of turbidity of the solution; after that, it was replaced with fresh PBS to continue the immersion; in addition, the change of the conductivity of PP@FSB was measured by the electrochemical workstation at each time point.

### Mechanical Tests

The mechanical properties of samples were evaluated by a universal testing machine. Rectangular samples of the swim bladder were cut and subjected to a single tensile test at a tensile rate of 2 mm min^−1^, and tubular samples of the swim bladder were created and subjected to single and cyclic compression tests at a compression rate of 0.5 and 1 mm min^−1^, respectively.

### Cytocompatibility Test

Cell activity was assessed using a CCK‐8 kit. Sterilized samples were incubated with PC12 cells, SCs, and DRG neurons in 96‐well plates. At the selected time points, a mixture of CCK‐8 reagent and culture medium was added to the plate at a ratio of 1:10 and incubated at 37 °C for 2 h. Next, the medium was moved to another 96‐well plate, and a microplate reader was utilized for absorbance detection.

### DRG Culture

DRGs were isolated from embryonic day 12–14 Sprague‐Dawley rats and collected in the culture medium in accordance with the previously established protocol.^[^ ^17^ ^]^ The swim bladder samples with different treatments were immersed in 75% ethanol solution for 1 h and exposed to ultraviolet light for sterilization. Following three washes with sterile PBS, the samples were transferred to a 24‐well plate, where 400 µL purification medium A containing 2 vol.% B27 neural supplement, 2 mm glutamine, 50 ng mL^−1^ nerve growth factor (NGF), 10 µm cytarabine, 1 vol.% penicillin‐streptomycin (PS) solution, and neurobasal medium, was added to each well. One DRG was then inoculated into the center of each sample and cultured in a cell incubator at 37 °C for one day. The next day, the culture supernates were replaced with purification medium B containing 2 vol.% B27 neural supplement, 2 mm glutamine, 50 ng mL^−1^ NGF, 10 µm uracil, 10 µm 5‐fluorouracil, 1 vol.% PS, and neurobasal medium, and incubated for one day. Finally, DRGs were cultured with a maintenance medium containing 2 vol.% B27 neural supplement, 2 mm glutamine, 50 ng mL^−^^1^ NGF, 1 vol.% PS solution, and neurobasal medium for two days.

### Animal Experiments

The Animal Ethics Committee of Zhongda Hospital, Southeast University School of Medicine, approved all animal experimental procedures (20230625001). Male adult Sprague‐Dawley rats, weighing ≈200–250 g, were selected to construct a sciatic nerve injury model. The rats were divided into five groups, namely Autograft, FSB, PDA@FSB, PP@FSB, and IKPP@FSB groups. Prior to in vivo implantation, a swim bladder was cut into a rectangular slice and curled into the tube using a capillary glass tube as a rolling template, to which the seam was fixed with sutures. The rats were anesthetized with isoflurane during the operation, and the sciatic nerves of the left leg were fully exposed. Then, a 10 mm‐long nerve segment was removed, and nerve bridging was accomplished utilizing various sample materials. Finally, the muscle and skin incisions were closed sequentially with 6.0 sutures in all groups. Following a 2‐month observation period, nerve regeneration and functional recovery were evaluated in each group.

### Statistics

Data analysis was performed using Origin 2021 software. The statistical data was presented as mean ± SD, with n = 3 unless otherwise specified. One‐way ANOVA followed by Tukey's test was used for statistical analysis, and statistical significance was defined as a *p*‐value of less than 0.05.

## Conflict of Interest

The authors declare no conflict of interest.

## Author Contributions

Y.Z. and R.C. conceived the idea. H.Z. designed and conducted the experiments and data analysis. H.W., D.X., B.Z., and X.L examined the data. H.Z. wrote the manuscript. Y.Z., H.W., M.L., C.Z., and D.X. revised the manuscript.

## Supporting information

Supporting Information

## Data Availability

The data that support the findings of this study are available from the corresponding author upon reasonable request.
