# Supplementary material for: PEDOT‐Integrated Fish Swim Bladders as Conductive Nerve Conduits
Source: Adv Sci (Weinh). 2024 Jun 17;11(31):2400827. doi: 10.1002/advs.202400827 (PMC11336940; doi:10.1002/advs.202400827)
Supplement: Supplementary file 1 — Supporting Information [file ADVS-11-2400827-s001.pdf]

ADVANCED SCIENCE
Open Access

Supporting Information

for *Adv. Sci.*, DOI 10.1002/advs.202400827

PEDOT-Integrated Fish Swim Bladders as Conductive Nerve Conduits

Hui Zhang, Dongyu Xu, Bin Zhang, Xiaofan Li, Minli Li, Chen Zhang*, Huan Wang*, Yuanjin Zhao* and Renjie Chai**

Supporting Information

PEDOT-integrated fish swim bladders as conductive nerve conduits

Hui Zhang, Dongyu Xu, Bin Zhang, Xiaofan Li, Minli Li, Chen Zhang*, Huan Wang*, Yuanjin Zhao*, Renjie Chai**

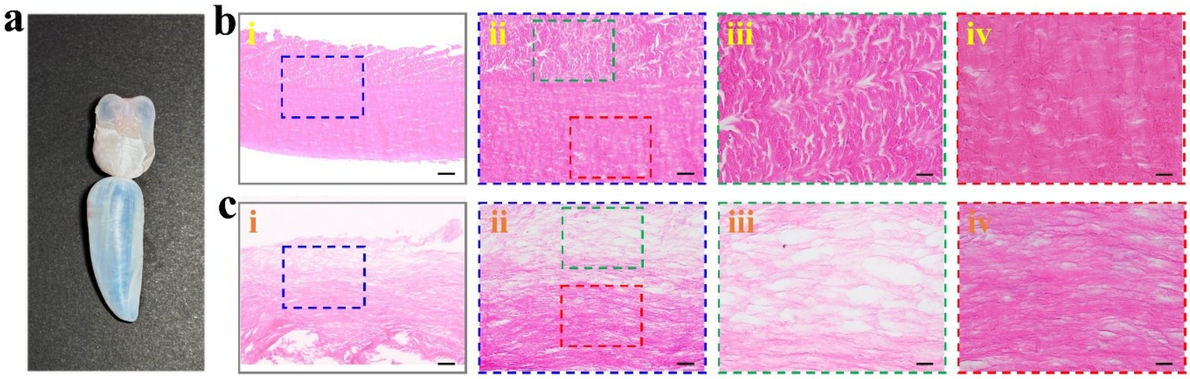

Figure S1. (a) Real images of native FSB. (b) H&E staining of UD-FSB. Scale bars are 100, 50, 20, and 20 μm, respectively. (c) H&E staining images of decellularized FSB. Scale bars are 100, 50, 20, and 20 μm, respectively.

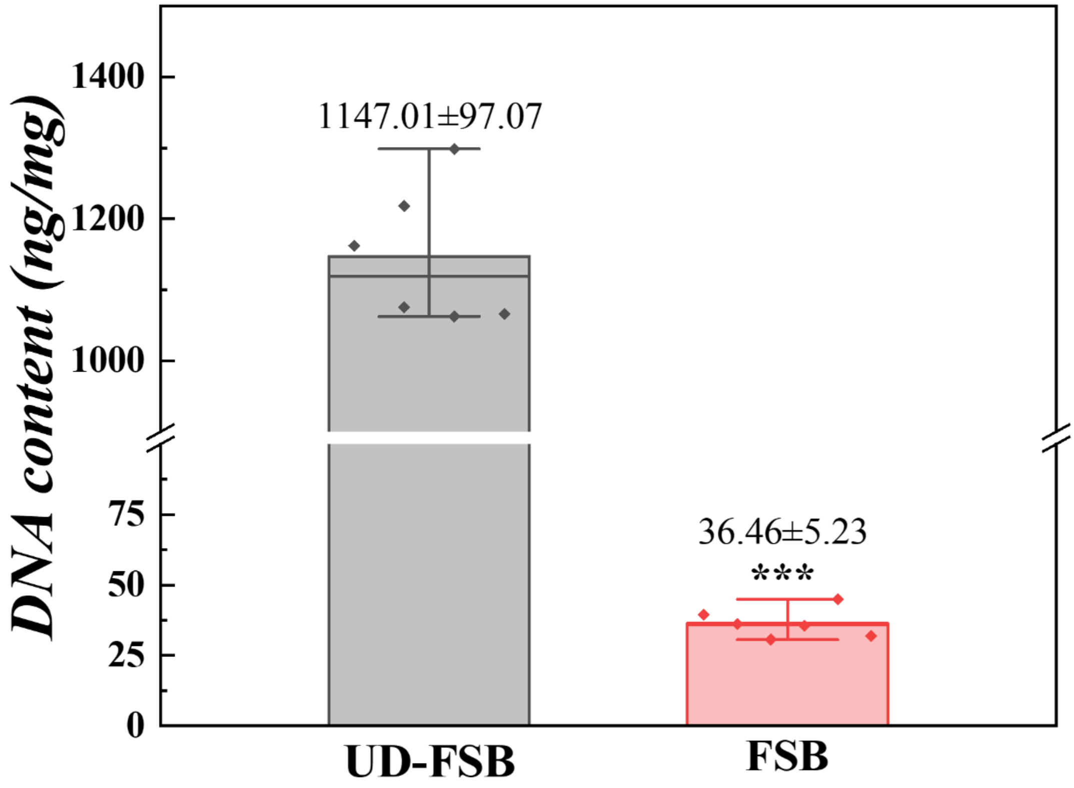

Figure S2. DNA content detection of UD-FSB and FSB.

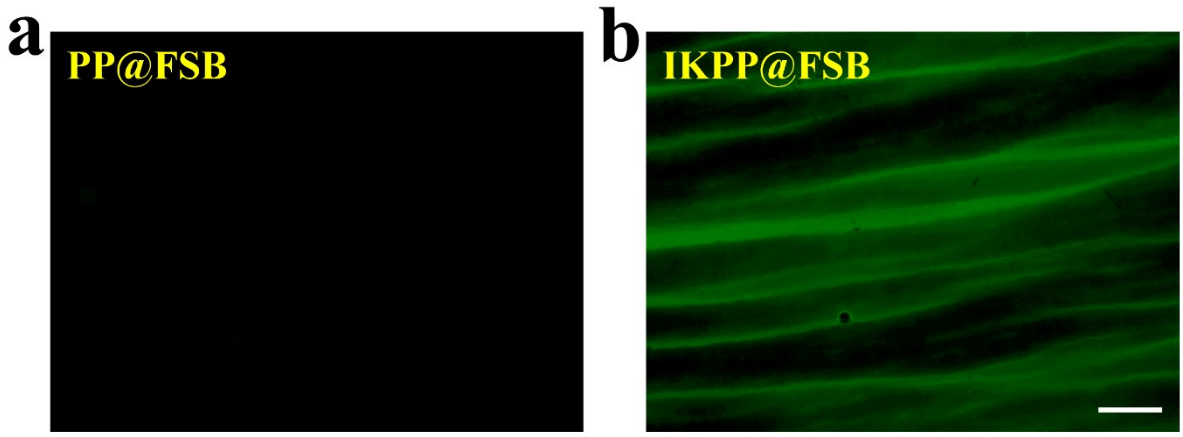

Figure S3. (a, b) Fluorescent images of PP@FSB (a) and IKPP@FSB (b). Scale bar is 200 μm.

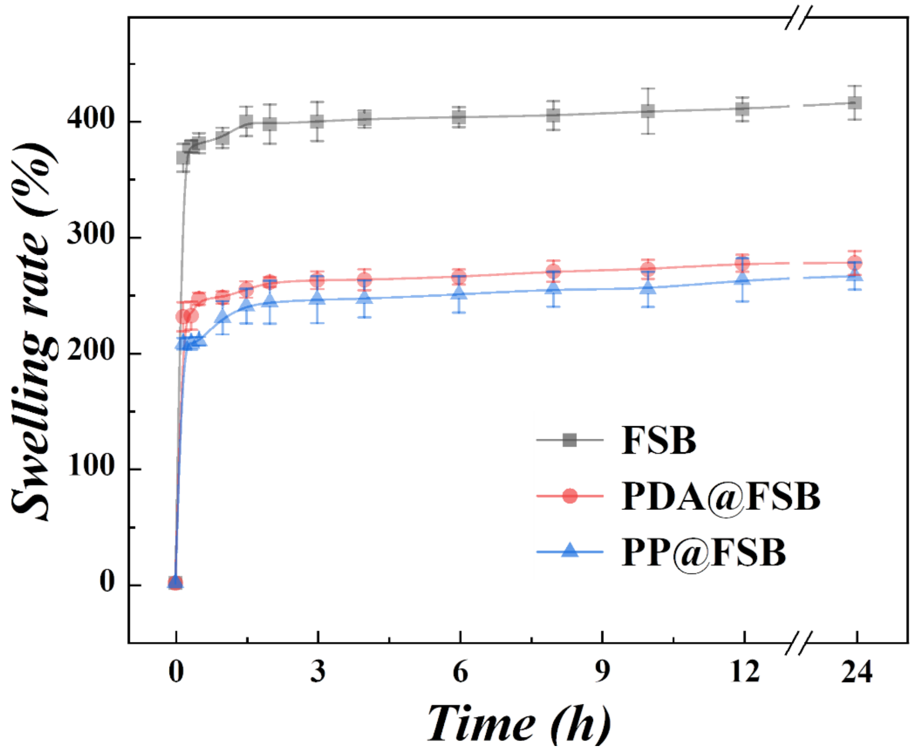

Figure S4. Swelling curves of FSB, PDA@FSB and PP@FSB.

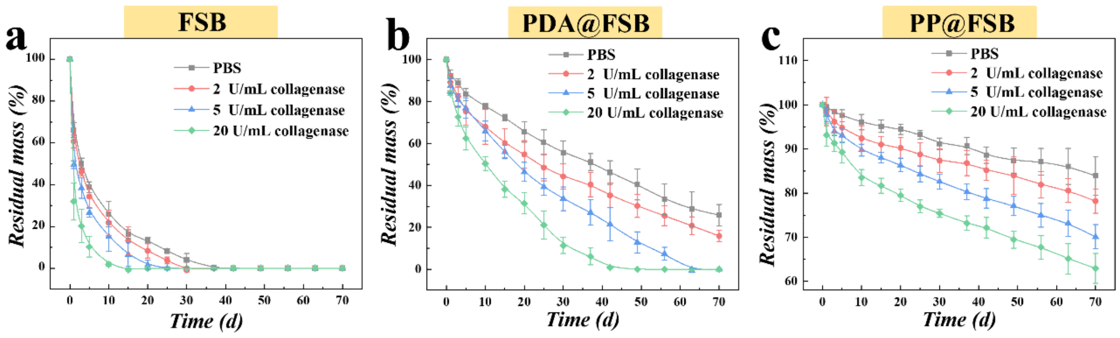

Figure S5. (a) Degradation curves of FSB in PBS, as well as 2, 5, and 20 U/mL collagenase solution. (b) Degradation curves of PDA@FSB in PBS, as well as 2, 5, and 20 U/mL collagenase solution. (c) Degradation curves of PP@FSB in PBS, as well as 2, 5, and 20 U/mL collagenase solution.

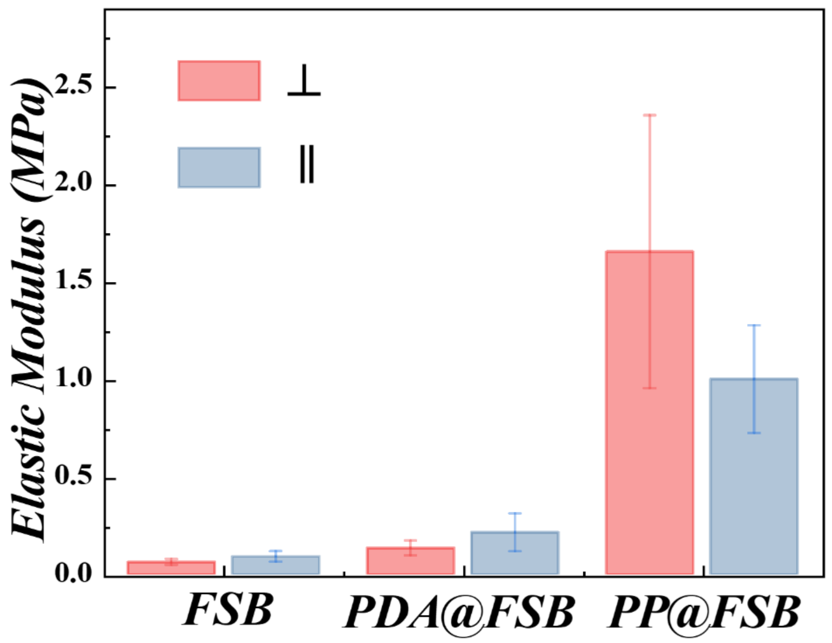

Figure S6. Elastic modulus of FSB, PDA@FSB and PP@FSB.

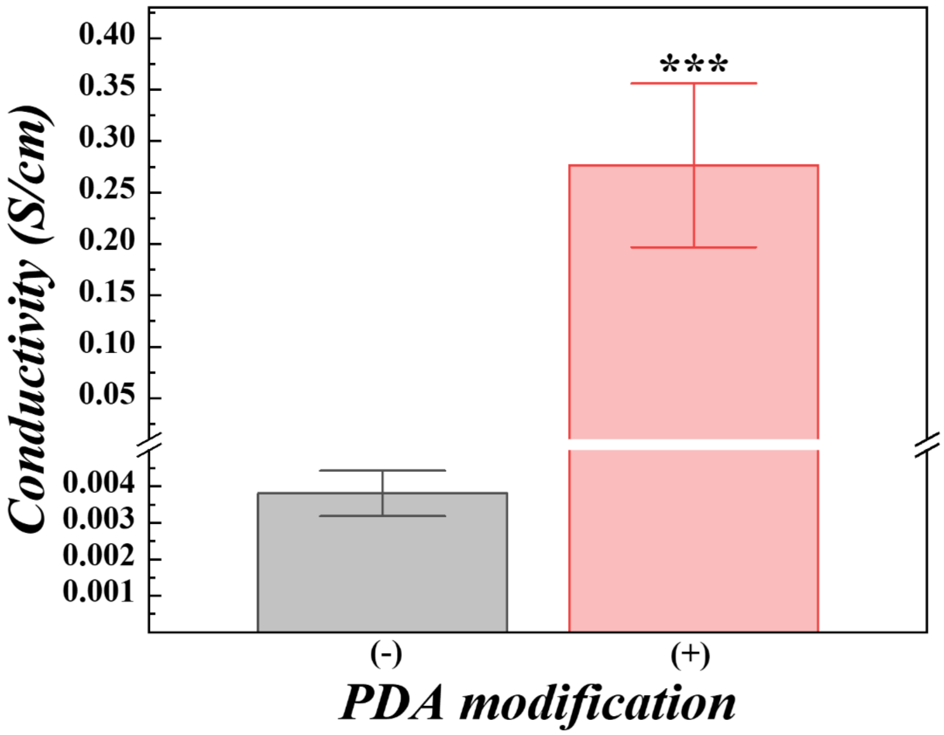

Figure S7. Conductivity of PP@FSB with or without PDA modification.

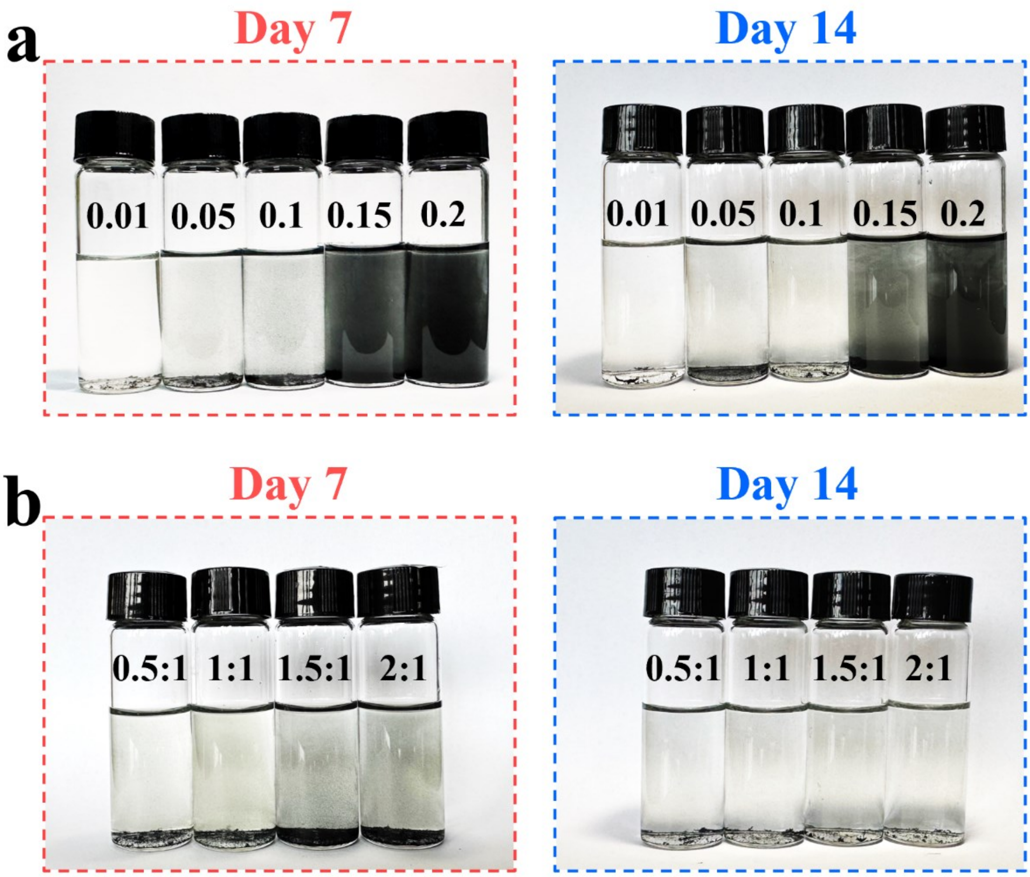

Figure S8. Stability of PEDOT conductivity in PBS solution.

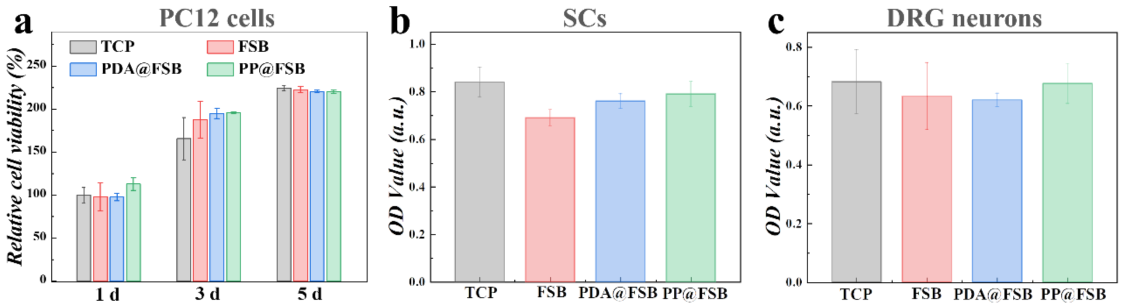

Figure S9. (a-c) CCK-8 results of undifferentiated PC12 cells (a), SCs (b) and DRG neurons (c) growth on different substrates.

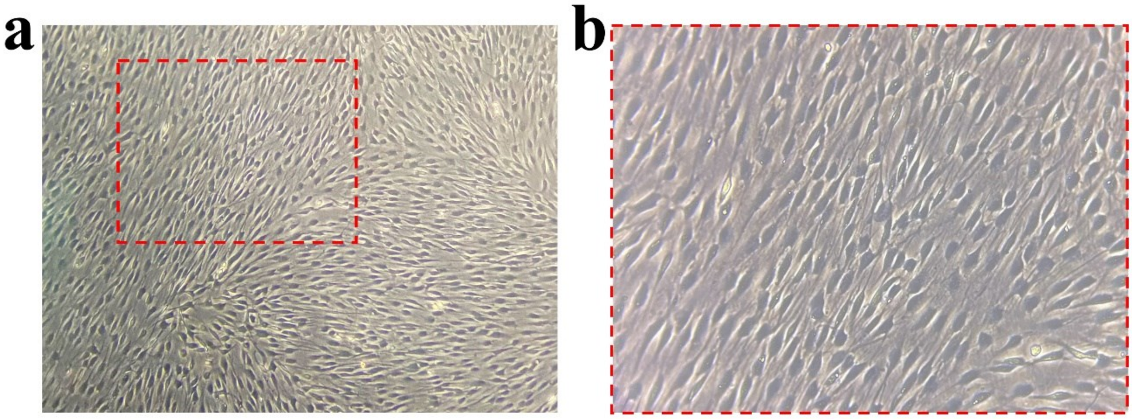

Figure S10. (a) Optical images of SCs. (b) Local enlarged images of (a).

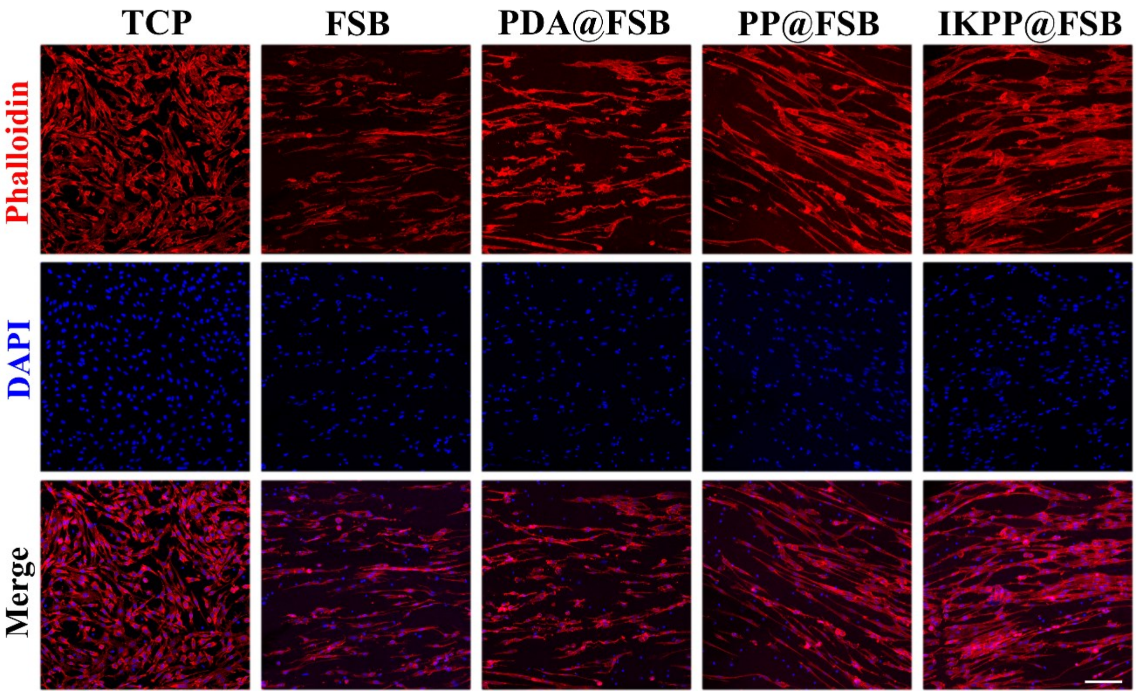

Figure S11. Immunofluorescent images of highly differentiated PC12 cells growth on different substrates. Scale bar is 80 μm.

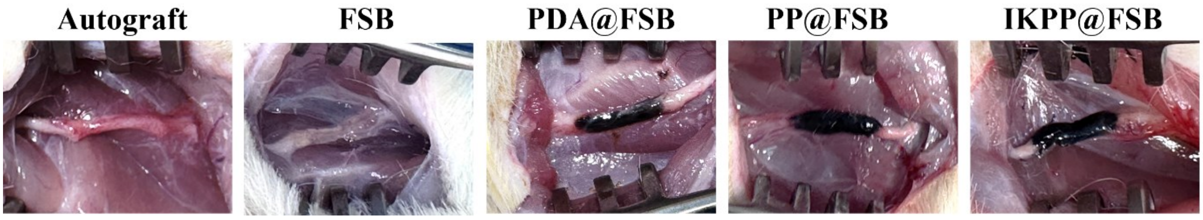

Figure S12. Representative images of regenerated nerves of rats with different treatments.

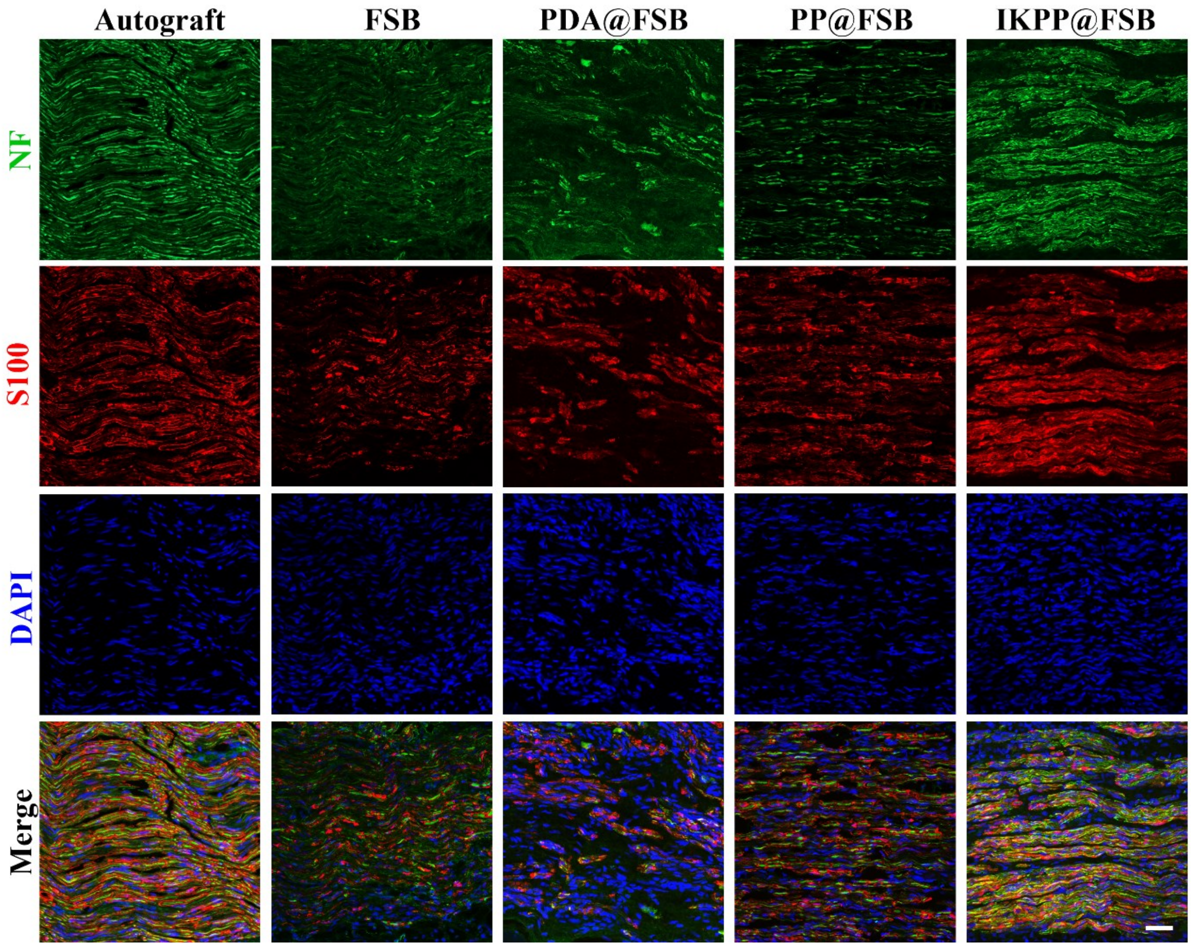

Figure S13. Immunofluorescent images of regenerated nerves of rats with different treatments. Scale bar is 10 μm.

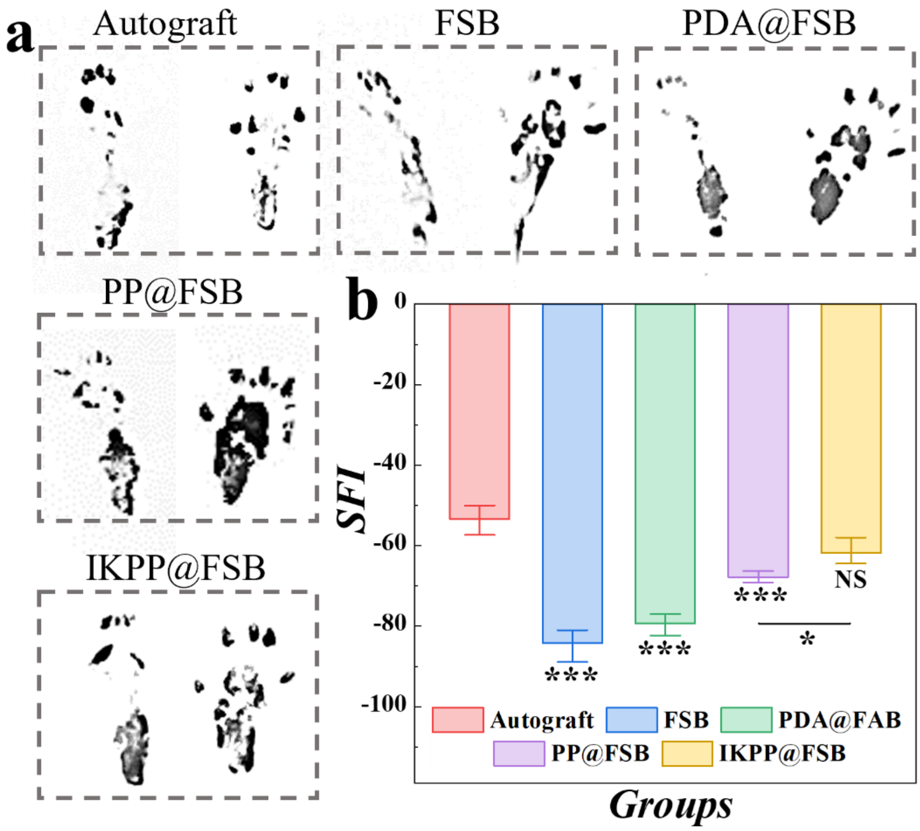

Figure S14. (a) Gait analysis of rats with different treatments. (b) SFI of rats with different treatments.
